# Morphological Transitions in Micelles of Amphiphilic Bottlebrushes upon the Adsorption and Compression at the Liquid Interface

**DOI:** 10.3390/polym14235076

**Published:** 2022-11-23

**Authors:** Alina S. Bugaeva, Rustam A. Gumerov, Igor I. Potemkin

**Affiliations:** Physics Department, Lomonosov Moscow State University, Leninskie Gory 1-2, 119991 Moscow, Russia

**Keywords:** bottlebrush polymers, self-assembly, liquid interface, adsorption, compression

## Abstract

Densely grafted comb-like macromolecules (bottlebrushes) with alternating solvophobic and solvophilic side chains were studied in a selective solvent and at the liquid interface using mesoscopic computer simulations. The effects of backbone length and copolymer composition were considered. While self-assembly in solution revealed only spherical aggregates for all ar-chitectures studied, adsorption onto the liquid interface in particular cases resulted in morpho-logical changes, with worm-like aggregates or a continuous monolayer observed. In turn, the compression of macromolecules at the interface also leads to morphological transitions, includ-ing the formation of a mesh-like percolated structure. The obtained results may be useful for the preparation of solid nanoparticles of anisotropic shape or nanostructured ultra-thin copolymer films.

## 1. Introduction

Block copolymers are a well-known type of macromolecule capable of self-assembly in melts and solutions [1], which can be employed in a wide range of possible applications [2]. In general, the morphology of resulting nanostructures depends on block compatibility, their solubility in the solvent, and copolymer composition. At the same time, a macromolecular architecture also greatly influences the behavior of copolymer systems. Particularly, the use of bottlebrush copolymers consisting of multiple side chains densely grafted to a long linear backbone allows obtaining large domain structures in bulk or monodispersed micelles in a selective solvent even at very low dilutions [3]. Another example exploiting the features of branched architecture is the formation of unimolecular aggregates of cylindrical shape [4], which distinguishes such macromolecular objects from their linear analogues. Overall, the bottlebrush copolymers can be used for the fabrication of anisotropic nanoparticles [4], nanotubes [5], photonic crystals [6], and nanoporous materials [7].

Along with the melt and solution, the self-assembly of block copolymers also occurs at liquid (oil-water [8] or air-water [9]) interfaces with the formation of ultra-thin films. The latter is of particular interest as the interfacial morphologies will not only depend on the aforementioned factors but also on the interactions between the blocks and solvent with the second phase. Adsorption at the interface produces nanostructures that are similar to those obtained in bulk or thin films for both linear [10,11,12] and non-linear macromolecules [13]. Simultaneously, these morphologies can be significantly altered by changing the surface pressure (such as, using the Langmuir–Blodgett technique [9,11,13]). For instance, the transition from dots to strands and then to a continuous monolayer has been demonstrated for polystyrene-*b*-poly(2-vinylpyridine) block copolymers at the air-water interface [9]. Meanwhile, the transition from ribbon-like to island-like morphology was observed for bottlebrush block copolymers with long polystyrene side chains [13]. Nevertheless, the behavior of bottlebrush copolymers at the liquid interface remains poorly understood. Currently, there are only a few publications considering their interfacial self-assembly [13]. Due to the branched architecture, the copolymer composition of brush macromolecules can be tuned to a great extent, i.e., by changing the lengths and the sequence of chemically distinct side chains along the backbone as well as the composition of the side chains themselves. Thus, one may expect the emergence of novel morphologies under interfacial confinement. 

Aside from the experiments, which are often a complex process, the computer simulations can be used to shed light on the features of bottlebrush copolymers and predict the new properties of such macromolecules [14,15,16,17,18,19,20]. In particular, it was found that the aggregation of asymmetric amphiphilic brushes in solution gives a rich variety of micellar morphologies depending on side chain compositions and grafting density [14]. Moreover, the possibility of the emergence of single-polymer complex nanostructures such as double, triple, and toroidal micelles through the rapid decrease in solvent quality has been shown for amphiphilic mikto-grafted bottlebrushes [16]. Finally, simulations of branched macromolecule self-assembly with polylactide and polystyrene side chains revealed that brushes with blocky side chain sequences form larger and more well-defined micelles by association with spatially segregated polystyrene side chains than brushes with random side chain sequences [18].

In this work, we aim to study the self-assembly of amphiphilic bottlebrushes with alternating solvophilic and solvophobic side chains at the liquid interface by means of computer simulations. We will show how the parameters of brush architecture, namely, the backbone and side chain lengths and the fraction of solvophobic side chains, influence the interfacial behavior of brush macromolecules. In addition, we will study the effect of uniaxial compression on the morphology of the monolayer formed by the bottlebrush copolymers.

## 2. Model and Simulation Method

*Method.* Dissipative particle dynamics (DPD) simulations [21,22] were used to study the behavior of bottlebrush copolymers [14,16,18,20]. Within the standard DPD framework, all polymer segments and solvent molecules are represented in terms of spherical beads of equal mass *m*, with each bead typically consisting of a group of atoms. The beads interact with each other by a pairwise additive force:

(1) $$F_{i}=\sum_{i\neq j}(F_{ij}^{C}+F_{ij}^{D}+F_{ij}^{R}+F_{ij}^{B})$$

where $F_{ij}^{C}$ is a conservative force responsible for the repulsion via soft potential characterized by the parameter *a_ij_* [21]. The bigger the value of *a_ij_*, the stronger the repulsion between the *i*th and *j*th beads. $F_{ij}^{D}$ and $F_{ij}^{R}$ are the dissipative and random forces, respectively, which serve as heat sinks and sources. $F_{ij}^{B}$ is a bonding force that keeps the beads in the polymer together. The sum runs among all the beads in the system. The first three terms in Equation (1) act only within a certain cutoff radius *r_c_,* which usually serves as the characteristic length scale unit. These terms are given by the following expressions:

(2) $$F_{ij}^{C}=\{\begin{array}{c} a_{ij}(1-r_{ij}){\bar{r}}_{ij},\\ \begin{array}{cc} & \begin{array}{cc} 0, & \end{array} \end{array} \end{array}\begin{array}{c} r_{ij}<1\\ r_{ij}\geq 1 \end{array}$$

(3) $$F_{ij}^{D}=-\lambda {[\omega (r_{ij})]}^{2}(v_{ij}\cdot {\bar{r}}_{ij}){\bar{r}}_{ij}$$

(4) $$F_{ij}^{R}=\sigma \omega (r_{ij})\xi_{ij}\Delta t^{-1/2}{\bar{r}}_{ij}$$

here ${\bar{r}}_{ij}=(r_{i}-r_{j})/r_{ij}$ is the unit vector pointing from the *j*th to the *i*th bead, $\omega (r_{ij})=(1-r_{ij})\;$ is a weight function that turns to zero when $r_{ij}\geq 1$, $v_{ij}=v_{i}-v_{j}$ is the relative velocity of the *i*th and *j*th beads, $\xi_{ij}$ is a zero-mean normally distributed random variable, and Δ*t* is a simulation timestep.

The bond $F_{ij}^{B}$ force is described by the harmonic potential:

(5) EB=12ks(r−r0)2

with specified spring constant *k_s_* and equilibrium bond length *r*_0_.

In the classic DPD approach, the momentum for each pair of beads is preserved. To satisfy the fluctuation–dissipation theorem, a relation $\sigma^{2}=2k_{B}T\lambda$ must be provided [22]. The value of *λ* is set to 4.5 for a reasonable rate of temperature equilibration. The evolution of the system is described by *N* equations of motion expressed through Newton’s second law, $m\;dv_{i}/\;dt=F_{i}$. All quantities are measured in units of the mass of the bead, *m*, thermal energy, *k_B_T*, and the cutoff radius of the interaction potential, *r_c_*. For convenience, the quantities are fixed as *m* = *k_B_T* = *r_c_* = 1, so that the characteristic timescale is defined as *τ* = *r_c_* (*m*/*k_B_T*) ^1/2^ and also equals 1 [22]. By setting the standard value of the number density *ρ* = 3, the interaction parameters *a_ij_* (in units of *k_B_T/r_c_*) can be mapped onto the Flory–Huggins parameters $\chi_{ij}$ by a linear relation [22]:

(6) $$a_{ij}\approx a_{ii}+3.27\chi_{ij}$$

where *a_ii_* = 25 for any two beads of the same type [22]. Finally, the backbone and the side chains are considered to be fully flexible, so we choose the following values for each bond: *k_s_* = 100 and *r*_0_ = 0.7. Additional details can be found elsewhere [21,22].

**Figure 1:**
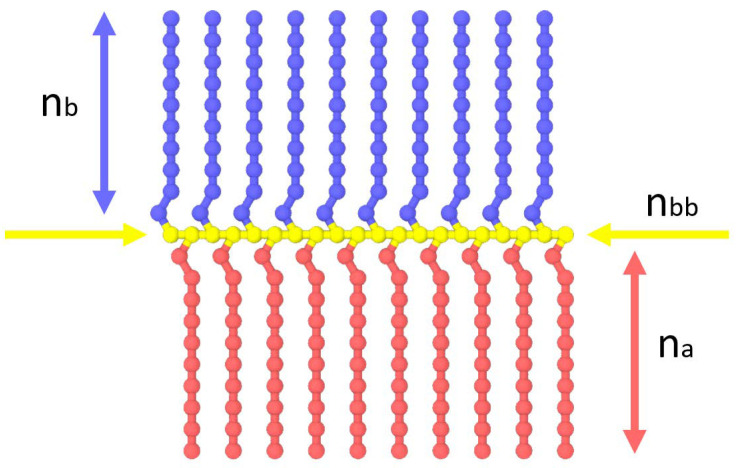
Schematic illustration of the studied amphiphilic bottlebrush copolymers with a homopolymer backbone (yellow) and alternating side chains composed of solvophilic A (red) and solvophobic B (blue) segments. For the current model, the fractions of A and B beads are equal (*f_a_* = 0.5).

*Models*. Bottlebrush copolymers were designed in the following way. Homopolymer side chains consisting either of segments of type A (solvophilic) or type B (solvophobic) were alternatingly attached to a backbone of type C (Figure 1). The number of segments in the backbone *n_bb_* varied between 20 and 200, while the number of segments in the side chains of type A was fixed at *n_a_* = 10. The length of the solvophobic blocks, *n_b_*, was then varied from 3 to 20. The fraction of side chains of type A – *f_a_* – was also varied. As a result, the overall solvohpilic:solvophobic ratio *n_a_* × *n_bb_* × *f_a_*:*n_b_* × *n_bb_* × (1-*f_a_*) varies between 1:1 and 1:4. The full list of considered brush architectures can be found in Table 1.

*Simulation Systems.* The simulation systems include polymer beads as well as solvent beads of types W and O, which correspond to nominal amounts of water and oil, respectively. The liquids are chosen as being strongly incompatible with each other; *a_OW_* = 80. The first solvent is good for A segments (*a_W_* = 25); poor for B segments (*a_BW_* = 50); and moderately poor for backbone beads (*a_CW_* = 30). The second phase is considered a poor solvent for all types of polymers (*a_AO_* = *a_BO_* = *a_CO_* = 50) and can also be considered an air environment for the current systems [17].

All simulations were performed using the open-source software LAMMPS [23], with the integration time step Δ*t* = 0.02*τ*, where τ is the characteristic timescale. There are two types of systems in our study. For the first type, the beads are placed in the simulation box with periodic boundary conditions in the *xy* plane and with dimensions *L_x_* = *L_y_* = 60*r_c_* and *L_z_* = 80*r_c_*. The lower part (*L_z_* ≤ 60*r_c_*) is filled with a 3% polymer solution, and the upper part (*L_z_* > 60*r_c_*) is filled with oil (air) beads. Initially, these systems were equilibrated for 5 × 10^6^ timesteps. Furthermore, the implicit reflective walls were placed at the top of the lower part of the box to prevent polymer adsorption at the liquid interface. The implementation of the walls had no effect on the characteristics of the polymer’s behavior in solution, and the thermodynamic parameters of the system were kept at constant values. After that, the walls were removed, and the adsorption of bottlebrush macromolecules was performed for an additional 10 × 10^6^ timesteps. For the second type of system, the uniaxial compression of polymer ensembles at the interface was considered. The macromolecules were placed in a large simulation box with periodic boundary conditions and with dimensions *L_x_* = *L_y_* = 100*r_c_* and *L_z_* = 30*r_c_*. After equilibration for 2 × 10^6^ steps, the systems were compressed along the x-axis while preserving their volume (at the fixed value of *L_y_* and the gradual increase in *L_z_*). *L_x_* was decreased in a stepwise manner until the value of *L_x_* = 30*r_c_* was reached. The decrease proceeded each 1 × 10^5^ timesteps followed by the additional 5 × 10^5^ steps to collect statistics and snapshots. Depending on the overall density of the monolayer, the compression step size ranged from 5 to 10*r_c_*.

## 3. Results and Discussion 

Amphiphilic bottlebrushes were simulated in solution and at the interface. In a selective solvent, all polymers were found to form spherical aggregates, and their mean aggregation numbers *N_mol_* and *N_agg_* (in terms of macromolecules and beads, respectively) were calculated [20] (Table 1). The following conclusions can be made: (i) an increase in backbone length decreases the aggregation number, which, for instance, fully agrees with the experimental studies by Hirai et al. for amphiphilic copolymers with long pendant groups acting as short side chains [24]; (ii) a decrease in the fraction of solvophilic branches increases the aggregation number even if the solvophilic/solvophovic ratio is close to the symmetric one (the first row in Table 1). Interestingly, even for the lowest value of the latter parameter, the aggregation number is close to unity, which is also seen for most cases of bottlebrush architectures. This is due to the high density of the side chains, resulting in the formation of unimolecular micelles.

The observed behavior can be explained as follows: The equilibrium size of the spherical micelles on the basis of block copolymers in a selective solvent is primarily determined by the ratio of volumes of the dense core and swollen corona (like the relative length of solvophobic and solvophilic blocks in the case of diblock copolymers). Variation in the backbone length practically does not change the ratio of solvophobic and solvophilic blocks. Therefore, a decrease in the backbone length should increase the aggregation number to keep the “optimum” size of the micelle. A decrease in the fraction of the solvophilic branches weakens their repulsion from each other and stimulates further aggregation of insoluble blocks until this aggregation is balanced by the stretching of the corona-forming blocks. 

*Adsorption.* As mentioned above, in this work, the main attention is paid to the adsorption of particles formed by the bottlebrush copolymers and their further compression. Thus, our first goal was to find out how the architectural parameters of the macromolecules affect the morphology of adsorbed aggregates. As was already stated, the initial morphology of the solution turned out to be the same for all cases.

**Figure 2:**
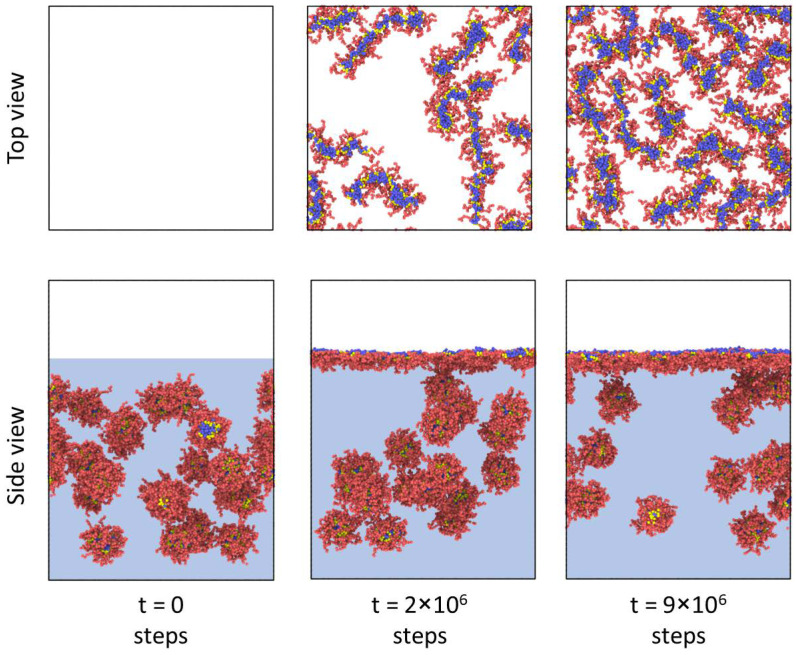
Snapshots depicting an amphiphilic bottlebrush solution at various stages of adsorption to the liquid interface. The macromolecular architecture parameters are: *n_b_* = 3, *n_bb_* = 100, and *f_a_* = 0.5. For convenience, the solvent beads are not shown.

Figure 2 depicts snapshots of an amphiphilic bottlebrush solution at various stages of adsorption to the liquid interface. The reason for this process is a decrease in the surface tension of the interface, similarly to other amphiphilic polymers [25]. At the same time, as mentioned above, if we observe spherical micelles in solution, then during adsorption we can see that the micelles “unfold” and gain a worm-like morphology. Thus, we can say that, in this case, adsorption to the interfacial boundary leads to a morphological transition, which is usually not observed for the case of linear block copolymers [9]. The physical reason for this phenomenon is the competition between interfacial energy, conformational entropy of branched macromolecules, and solvophobic interactions between B blocks and solvents. On the one hand, the high interfacial energy (*a_WO_* = 80 or *χ_WO_* ≈ 17) makes macromolecule adsorption with insoluble block localization at the very interface energetically favorable. Simultaneously, due to the peculiarities of polymer architecture, the conformational entropy of the side chains is lower than for linear copolymers of a similar composition, and, therefore, the preservation of the spherical morphology during adsorption would lead to a strong loss in the entropy of the swollen blocks. Thus, the shape of the adsorbed brushes becomes more non-spherical. This effect is comparable to the adsorption of homopolymer bottlebrushes on a solid substrate, which results in a significant increase in the macromolecule’s persistent length [26,27].

**Figure 3:**
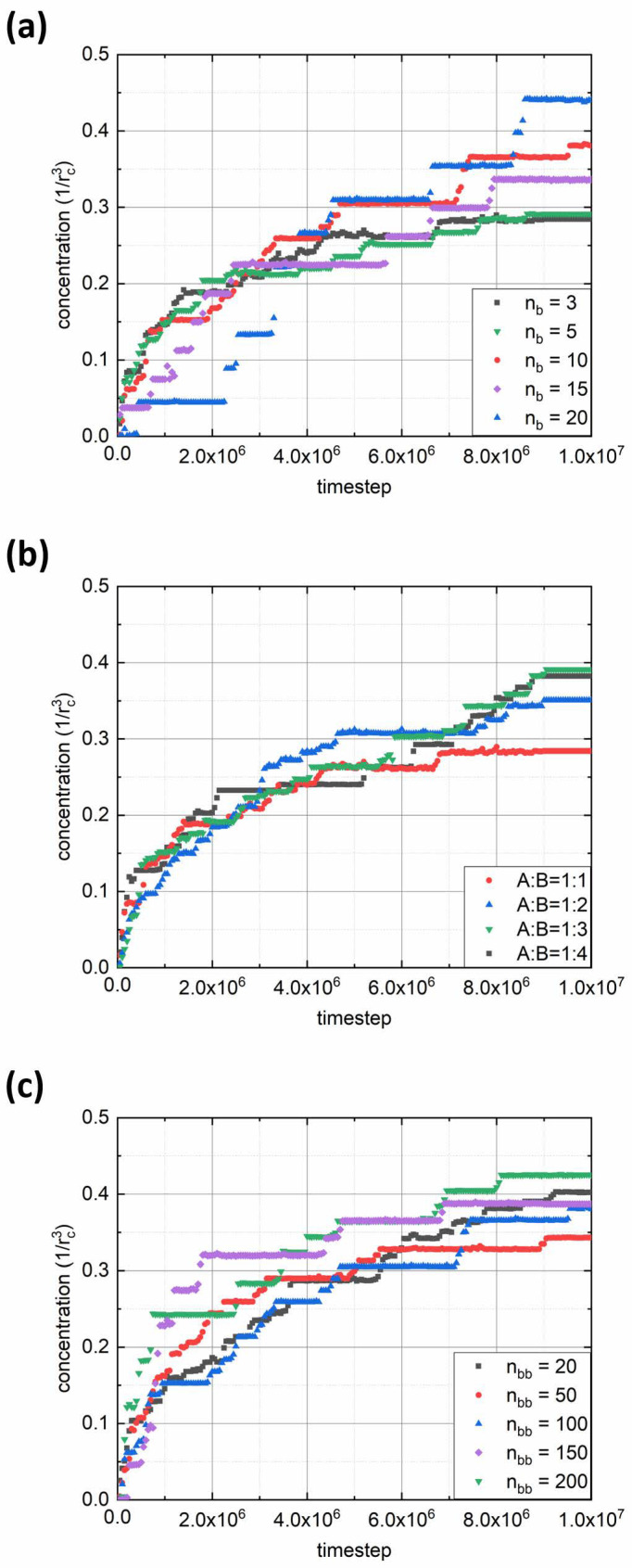
Evolution of polymer concentration at the liquid interface over time. Each graph depicts the effects of different parameters of the macromolecular architecture: (**a**) solvophobic side chain length (*n_bb_* = 100 and *f_a_* = 0.5); (**b**) ratio between solvophilic and solvophobic side chains A:B (*n_b_* = 3 and *n_bb_* = 100); and (**c**) backbone length (*n_b_* = 10 and *f_a_* = 0.5).

We performed a quantitative analysis of the adsorption rate by calculating the polymer concentration in the interface region with thickness *d* = 2*r_c_*. Figure 3 shows plots of polymer concentration in the interfacial layer versus simulation time for various parameters of the bottlebrush architecture: solvophobic chain length *n_b_* (Figure 3a), fraction of solvophilic side chains expressed through the ratio between numbers of solvophilic and solvophobic grafts *f_a_*:(1-*f_a_*) = A:B (Figure 3b), and backbone length *n_bb_* (Figure 3c). From the first graph, it can be seen that for short solvophobic chain lengths (*n_b_* = 3, 5), the polymer concentration at the interface grows fast at the beginning and then reaches a plateau toward the end due to steric repulsion between adsorbed solvophilic side chains. In addition, the reason for fast interface saturation may lie in morphological transitions in adsorbed aggregates (see below). On the contrary, for macromolecules with longer B-type side chains (*n_b_* ≥ 10), the concentration begins to increase noticeably towards the end of the considered simulation interval. This is due to the fact that an increase in *n_b_* increases the aggregation number (Table 1) and the size of the aggregates formed, thus leading to a decrease in the diffusion coefficient [25]. The latter effect appears to be visible within the first 3 × 10^6^ simulation steps. At the same time, the longer the *n_b_*, the denser the monolayer at the end of the simulation run (regarding the fact of decreasing diffusion, see Figure 3a). In turn, the variation of the A:B ratio displays a rather opposite trend: the adsorption rate weakly depends on this parameter (Figure 3b). Such an effect is due to the interfacial aggregation of bottlebrush aggregates (see below), which is also seen by the obvious increase in final polymer concentration with the increase in the solvophobic side chain fraction. Finally, from the third graph, it can be seen that the backbone length has almost no effect on the adsorption rate (Figure 3c). Indeed, one may argue that the increase in *n_bb_* should increase the size of bottlebrush aggregates. On the other hand, since the aggregation number increases simultaneously (Table 1), the overall size of the aggregates will be similar regardless of backbone length. An exception can be made for the case where *n_bb_* = 200, where the final polymer concentration at the interface is the highest (Figure 3c), which is the result of bottlebrush topology.

**Figure 4:**
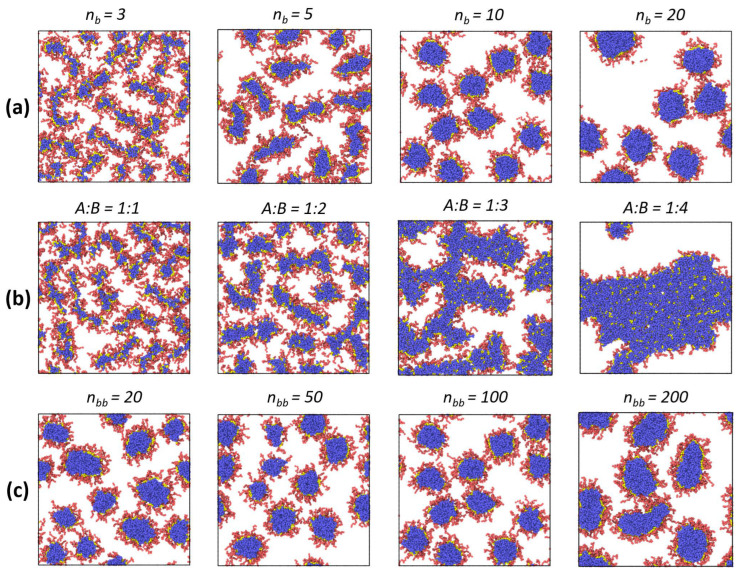
Top views of the liquid interface with adsorbed amphiphilic bottlebrushes at the end of the simulation run. Each row depicts the effects of different parameters of the macromolecular architecture: (**a**) solvophobic side chain length (*n_bb_* = 100 and *f_a_* = 0.5); (**b**) ratio between solvophilic and solvophobic side chain A:B (*n_b_* = 3 and *n_bb_* = 100); and (**c**) backbone length (*n_b_* = 10 and *f_a_* = 0.5).

Further on, we investigated the influence of the bottlebrush architecture on the final interfacial morphology. Figure 4a presents the top-view images of the liquid interface with the adsorbed macromolecules with different lengths of solvophobic side chains. It can be seen that morphological changes during adsorption are present only when the solvophobic chains are shorter than the solvophilic ones (*n_b_* = 3, 5), i.e., when the contribution from solvophobic interactions is sufficiently small. Otherwise, the spherical morphology is preserved. Nevertheless, no interfacial aggregation is observed (Figure 4a). Meanwhile, the composition of amphiphilic macromolecules with the chosen architecture can change not only due to the length of side chains but also due to the variation of the A:B ratio, which is shown in Figure 4b. Here, an increase in the proportion of solvophobic side chains leads to a thickening of the adsorbed micelles and the possibility of their branching (A:B = 1:2). In the limiting case, when the proportion of solvophobic chains is much larger, one can observe the formation of long and thick worm-like aggregates (A:B = 1:3) or a continuous monolayer (A:B = 1:4) due to interfacial aggregation. Finally, the effect of backbone length (which can be considered the effect of molecular weight at a fixed copolymer composition) is demonstrated in Figure 4c. As can be seen, at small values of *n_bb_*, the situation is closer to that of linear copolymers, and the bottlebrushes aggregate with each other (Table 1). As a consequence, the sizes of adsorbed micelles vary more than in the case of macromolecules with a longer backbone (*n_bb_* > 50). In the case of the longest backbone (*n_bb_* = 200), the shape of adsorbed aggregates can deviate from the spherical one, and for several macromolecules, a more ellipsoidal morphology can be found. Nevertheless, we may conclude that the morphology of surface micelles depends weakly on backbone length at the considered range of *n_bb_* values. 

**Figure 5:**
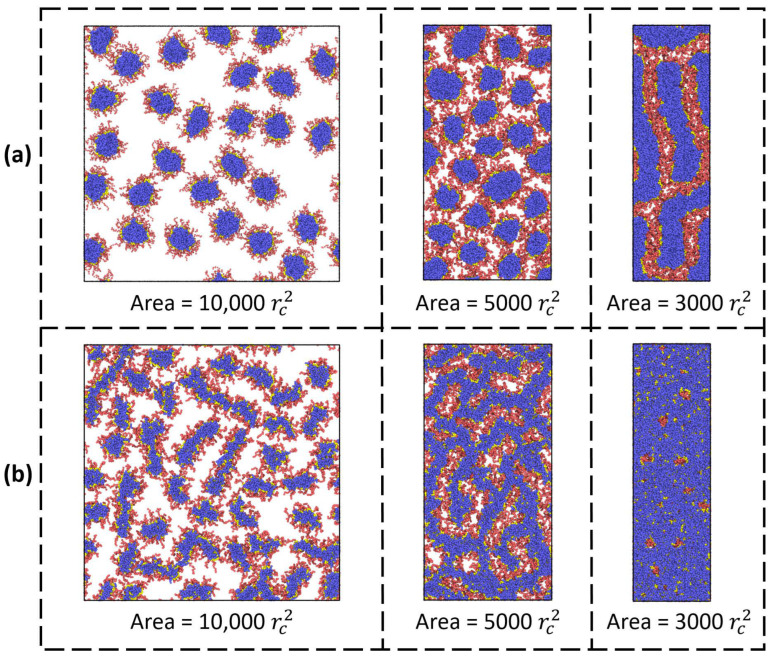
Top views of the liquid interface with adsorbed amphiphilic bottlebrushes at different compression ratios. The interfacial area is compressed from *L_x_* × *L_y_* = 100 × 100 to 50 × 100 and then to 30 × 100. The bottlebrush architecture parameters are: (**a**) *n_b_* = 10, *n_bb_* = 100, and *f_a_* = 0.5; and (**b**) *n_b_* = 3, *n_bb_* = 100, and *f_a_* = 0.33.

*Compression.* Let us now consider the effect of monolayer compression on the morphology of bottlebrush copolymers. Figure 5a presents the top view images of a monolayer at different compression ratios expressed through the total interfacial area *A* = *L_x_* × *L_y_*. The lengths and fractions of solvophilic and solvophobic side chains are equal, and the backbone length equals 100. It can be seen that the compression of the monolayer induces interfacial aggregation and the transition from spherical to cylindrical morphology. As in the case of micelles formed by linear diblock copolymers, this effect is explained by the orientation of the swollen side chains (the blocks) upon compression to the phase where their conformational entropy is higher. Indeed, compression controls the fraction of adsorbed solvophilic blocks: the higher the compression, the lower their fraction. For the case of diblock copolymers on a solid surface, it has been shown that mushroom-like micelles are favorable when the 2D blocks are long enough and a stripe-like structure is formed when the 2D blocks are short [28,29]. More interesting situations are observed when the length of solvophobic chains is short (*n_b_* = 3) and their proportion is twice that of solvophilic chains (Figure 4b). In this case, the initial worm-like morphology of macromolecules initially transforms into a continuous structure resembling the perforated lamellar and then into a continuous lamellar-like monolayer. 

**Figure 6:**
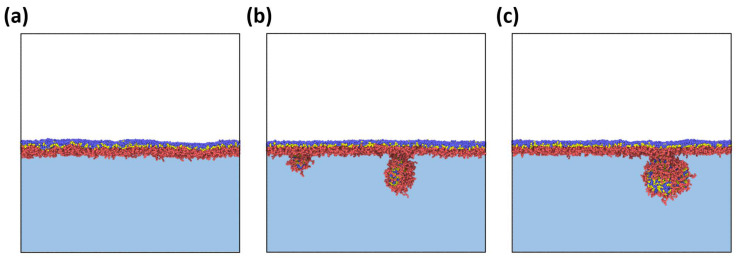
Side views of a strongly compressed bottlebrush monolayer at different simulation times: (**a**) right after the end of compression, t = 0 steps; (**b**) t = 1 × 10^6^ steps; and (**c**) t = 2 × 10^6^ steps. The interfacial area of *L_x_* × *L_y_* = 30 × 100. The bottlebrush architecture parameters are: *n_b_* = 3, *n_bb_* = 100, and *f_a_* = 0.2.

For both cases of bottlebrush architecture shown in Figure 5, no desorption of macromolecules from the boundary was observed, i.e., the surface energy of the boundary turned out to be higher than the elastic stress invoked in the monolayer due to compression. A different situation is observed in the case of a highly compressed monolayer (*L_x_* × *L_y_* = 30 × 100) formed by macromolecules with *n_b_* = 3, *n_bb_* = 100, and *f_a_* = 0.2 (Figure 6). Here, an initially strong undulation of the monolayer is followed by the desorption of part of the macromolecules from the boundary, i.e., the elastic limit of the monolayer turned out to be exceeded.

**Figure 7:**
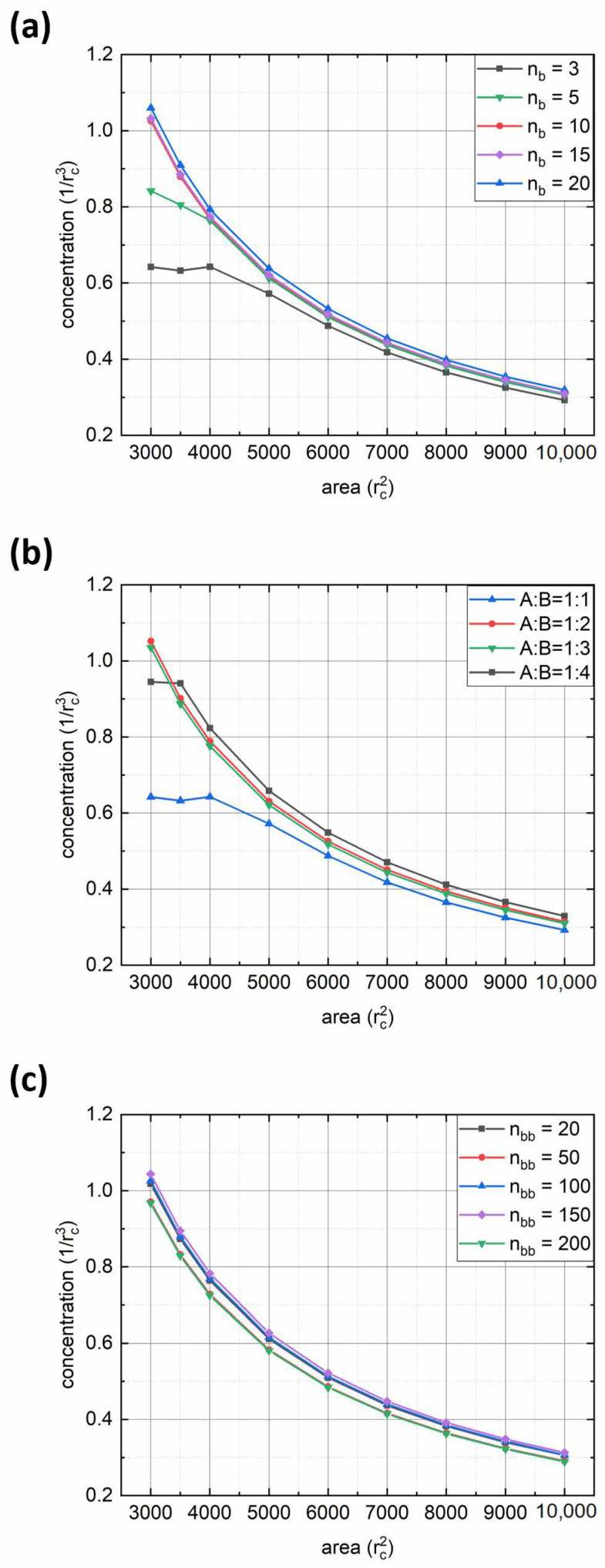
Dependences of polymer concentration in the monolayer on the interfacial area at different: (**a**) solvophobic side chain length (*n_bb_* = 100 and *f_a_* = 0.5); (**b**) ratio between solvophilic and solvophobic side chains A:B (*n_b_* = 3 and *n_bb_* = 100); and (**c**) backbone length (*n_b_* = 10 and *f_a_* = 0.5).

Let us carry out a quantitative analysis of the obtained results. Figure 7 shows the dependencies of polymer concentration in the monolayer on the interfacial area for various bottlebrush architectures. One can observe that in all cases, the concentration increases exponentially upon compression. However, several curves contain the inflection points, after which the exponential trend disappears, and the concentration reaches a plateau. These points can serve as an indicator of the monolayer’s elastic limit. Obviously, this limit depends on the fraction of solvophobic side chains and their lengths; the smaller the fraction and length, the more readily desorption occurs (Figure 7a,b). However, desorption may occur when the fraction of solvophilic side chains is small enough (*f_a_* = 0.2, black curve in Figure 7b). Simultaneously, there is no desorption for more solvophobic copolymers (*n_b_* ≥ 15, purple, and blue curves in Figure 7a). This is a direct consequence of bottlebrush topology. Finally, the backbone length appears to have no effect on the elastic limit of the monolayer, and all the curves in Figure 7c practically coincide.

**Figure 8:**
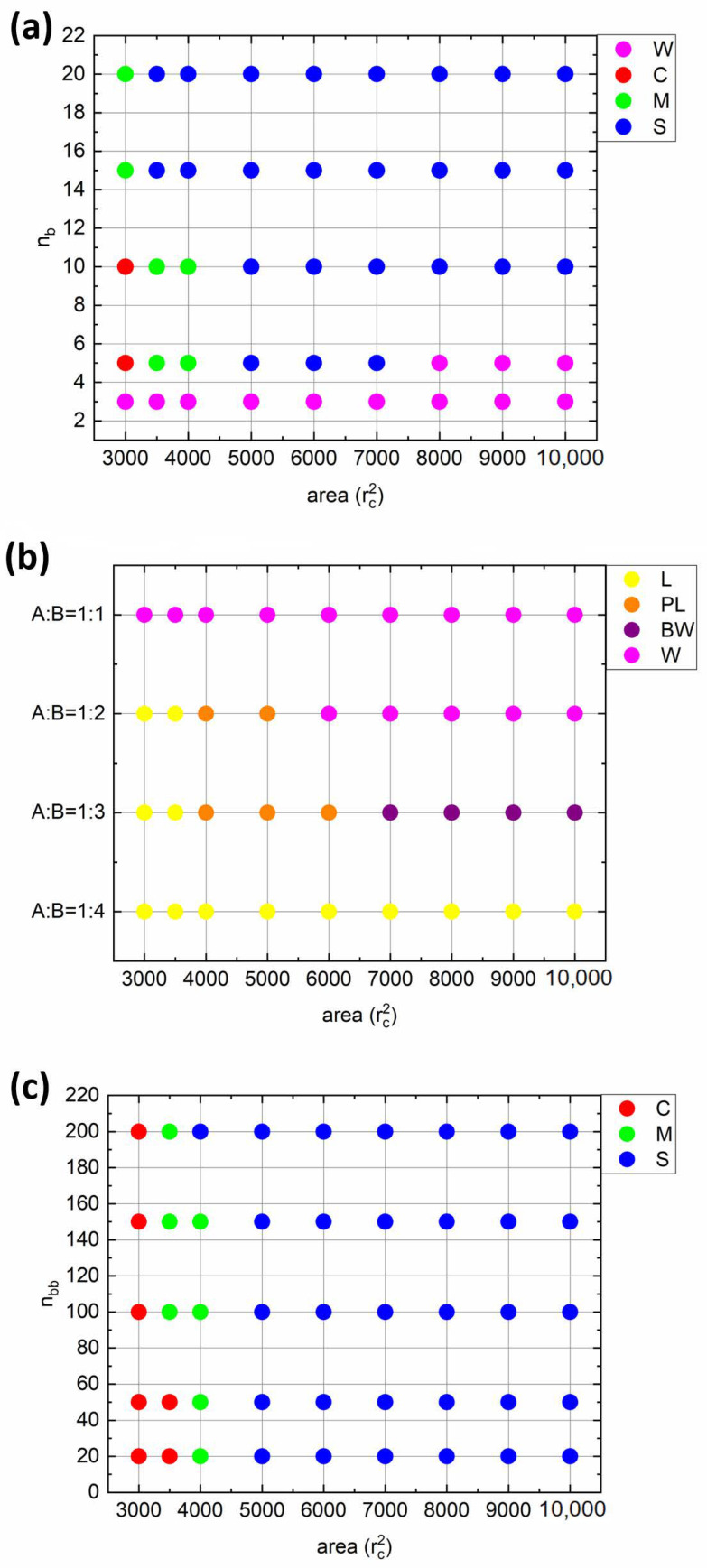
Diagrams of states of the interfacial bottlebrush monolayer depending on the degree of compression and (**a**) solvophobic side chain length (*n_bb_* = 100 and *f_a_* = 0.5), (**b**) ratio between solvophilic and solvophobic side chain A:B (*n_b_* = 3 and *n_bb_* = 100); and (**c**) backbone length (*n_b_* = 10 and *f_a_* = 0.5).

Finally, let us examine the morphologies of all considered monolayers by constructing the state diagrams (Figure 8). The following conclusions can be drawn. When the solvophobic side chains are short (*n_b_* = 3), the worm-like (W) morphology is observed at all compression ratios (Figure 8a). An increase in *n_b_* leads to the emergence of spherical (S) and cylindrical (C) morphologies at intermediate and high compression ratios, respectively (*n_b_* = 5; Figure 8a). In addition, a mixed state can be found at *A* = 4000*r_c_*^2^. The further increase in *n_b_* leads to the disappearance of the W morphology. On the other hand, when the fraction of solvophobic chains increases, upon compression, the worm-like aggregates begin to merge, forming a continuous perforated lamellar-like (PL) and then lamellar-like (L) morphology (A:B = 1:2, Figure 8b). The further shift in A:B ratio towards more solvophobic composition results in the formation of branched worm-like morphology (BW), which transforms into PL and then L at higher compression ratios (A:B = 1:3). When the amount of solvophilic side chains is minimal (A:B = 1:4), the monolayer morphology remains constant upon compression, though the monolayer is not continuous at high interfacial areas (as shown in Figure 4b). Finally, from the last diagram (Figure 8c), we can see that the increase in backbone length shifts the transition point from S to C morphology to slightly higher compression ratios (*n_bb_* = 20 vs. *n_bb_* = 200), while the overall morphological trend does not change at all. 

## 4. Conclusions

We have systematically studied the self-assembly of amphiphilic bottlebrush copolymers with alternating solvophilic and solvophobic side chains at the liquid interface. In particular, the behavior of macromolecules in a selective solvent was considered, as well as their adsorption to the interface followed by compression of the monolayer. The time- and compression-dependent polymer concentration functions for various bottlebrush architectures were plotted. The possibility of morphological transition of bottlebrush-based micelles during adsorption was shown. It has been found that the adsorption rate depends more on the length of solvophobic side chains and less on their fraction as well as the backbone length. Meanwhile, the compression of a monolayer also contributes to a change in the morphology. As a result, the diagrams of the states of the monolayer were constructed. For the considered compression ratios, the monolayer remains stable (no desorption occurs) when the length of solvophobic chains is equal to or greater than the length of solvophilic chains at equal side chain fractions. In turn, the desorption of macromolecules from the interface occurs when the length of solvophobic chains is less than the length of solvophilic ones. In addition, desorption was observed when the fraction of solvophilic chains was small enough. 

In the current work, we considered only macromolecules with alternating side chains of different sorts. Simultaneously, our research interests include a direct comparison with linear analogues as well as bottlebrush copolymers with blocky side chain sequences, which will be investigated further in the future.

We believe that our findings will encourage further experimental studies of bottlebrush systems (such as the those based on hydrophilic poly(ethylene oxide) and hydrophobic polystyrene [30] or poly(n-butyl)acrylate [31] with the possible addition of a volatile cosolvent to increase the mobility of the collapsed side chains [9]) which will eventually allow for the formation of ultra-thin polymer films with desired morphologies.

## Figures and Tables

**Table 1:** Characteristics of amphiphilic bottlebrushes in a selective solvent. For each case, the length of solvophilic branches *n_a_* = 10.

Backbone Length *n_bb_*	The Length of Solvophobic Branches *n_b_*	The Fraction of Solvophilic Branches *f_a_*	The Overall Solvophilic/ Solvophobic Ratio	Mean Aggregation Number (Macromolecules) *N_mol_*	Mean Aggregation Number (Beads) *N_agg_*
100	3	0.2	5:6	2.28 ± 0.15	548 ± 37
100	3	0.25	10:9	1.52 ± 0.10	342 ± 22
100	3	0.33	5:3	1.14 ± 0.00	230 ± 0
100	3	0.5	10:3	1.00 ± 0.00	150 ± 0
100	5	0.5	2:1	1.00 ± 0.00	250 ± 0
100	15	0.5	2:3	1.09 ± 0.02	817 ± 15
100	20	0.5	1:2	1.18 ± 0.00	1180 ± 0
20	10	0.5	1:1	3 ± 0.16	300 ± 16
50	10	0.5	1:1	1.34 ± 0.02	335 ± 5
100	10	0.5	1:1	1.08 ± 0.04	540 ± 20
150	10	0.5	1:1	1.00 ± 0.00	750 ± 0
200	10	0.5	1:1	1.00 ± 0.00	1000 ± 0.00
